# Quality attributes of date and wheat flour pineapple juice blended cookies as affected by different baking temperatures

**DOI:** 10.7717/peerj.14876

**Published:** 2023-02-22

**Authors:** Chigozie F. Okoyeuzu, Chioma N. Okoronkwo, Chinwendu R. Eze, Chisom V. Otuonye, Mouandhe Imamou Hassani, Onyekachukwu C. Nduka, Helen E. Ufondu, Charles Odilichukwu R. Okpala, John I. Eze

**Affiliations:** 1 Department of Food Science and Technology, University of Nigeria, Nsukka, Nsukka, Enugu State, Nigeria; 2 Institut National de Recherche Pour L’Agriculture, La Pêche et L’Environnement (INRAPE), Moroni, Comoros; 3 Faculty of Biotechnology and Food Sciences, Wroclaw University of Environmental and Life Sciences, Wroclaw, Poland; 4 UGA Cooperative Extension, College of Agricultural and Environmental Sciences, University of Georgia, Athens, Georgia, United States

**Keywords:** Moisture, Cookies, Date flour, Wheat flour, Pineapple juice, Energy value

## Abstract

Studying the effects of temperature on cookies are necessary especially if the impact on quality attributes are to be deduced. More so, blending wheat flour, date flour, and pineapple juice could improve the nutrient quality required in modern-day cookies. This current study investigated the quality attributes of date and wheat flour pineapple juice blended cookies as affected by different baking temperatures. With pineapple juice serving as water substitute, the formulated date, and wheat flour blends adhered to the following ratios: 100:0, 90:10, 80:20, and 70:30. Baking cookies involved two different temperatures (160 °C and 180 °C) with constant time (30 min). Quality attributes determined proximate composition, micronutrients, physical and functional properties, and microbial and sensory qualities. Cookies proximate results showed moisture (6.89–7.40%), protein (8.73–10.22%), fat (14.37–15.99%), fiber (1.02–1.11%), ash (0.77–1.20%) and carbohydrate (64.85–67.71%). Various ranges appeared, from energy values (434.90–444.10 kcal), minerals (calcium = 33.18–62.45 mg/100 g; iron = 3.47–5.75 mg/100 g; potassium = 100.07–358.63 mg/100 g), vitamins (vitamin A =1.99–4.89 mg/100 g; vitamin C = 0.04–0.15 mg/100 g), physical (weight = 7.4–7.75 g; diameter = 3.50–4.01 cm; thickness = 0.99–1.20 cm; volume = 3.11–3.77 cm^3^; density = 2.06–2.41 g/cm^3^; spread ratio = 2.92–4.05 cm^3^), to functional (water absorption = 1.14–1.18 g/g; oil absorption capacity = 1.31–1.33 g/g; bulk density = 0.74–0.76 g/mL) properties. The microbial loads seemed somewhat acceptable as overall acceptability favoured sample WDFb (90% wheat, 10% date flour). The acceptability of cookies baked at 160 °C over those baked at 180 °C suggests the need for further studies to determine the energy requirements, and long-term environmental implications such (baking) temperatures would pose.

## Introduction

Cookies refer to a baked product generally containing three major ingredients flour, sugar, and fat, which are mixed with other minor ingredients to form a dough. Cookies are among the best-known quick snack products (Farheena, Avneesh & Ugma, 2015), typical baked ready-to-eat snacks with attractive features like wide consumption, more convenience with long shelf-life, and promising vehicles for important nutrients (Ajibola, Oyerinde & Adeniyan, 2015). Cookies and other bakery products are increasingly becoming fast food products for every age group, easy to carry, of attractive taste, largely lowered cholesterol, as well as digestible and affordable (Farheena, Avneesh & Ugma, 2015). Cookies could be characterized by promising sugar and lower moisture, different from other baked foods like bread and cakes (Hanan & Al-Sayed, 2013). Main (cookies) ingredients include wheat flour, fat (margarine), sugar and water, milk, salt, aerating agent, emulsifier, flavour, *etc*., and further enriched or fortified with other ingredients to meet specific nutritional or therapeutic consumer (Ajibola, Oyerinde & Adeniyan, 2015). The stiffness and handling of the dough make the classification of cookies quite convenient. Largely understood, there are six basic types of cookies, namely: Drop, bar, rolled, refrigerator (icebox), pressed, and molded. The dough for bar cookies and drop cookies is softer than the dough for other types of cookies (Manley, 2001; Kieffer, 2020). The flour used in making cookies is basically from wheat or composite flour which forms the basic ingredients of bakery products including bread, rolls, cakes, cookies, and other bakery products (Giwa & Ikujenlola, 2010). Wheat is principally used in bakery products due to its appreciated rheological characteristics. Wheat flour is the basic structural component of most batter and dough products. It can perform these textural functions because of gluten content, which allows the expansion of air cells and provide rigidity after baking (Kulkarni & Joshi, 2013).

Date palm fruit (*Phoenix dactylifera* L.) locally called ‘debino’ in the Hausa language, from the family of Arecaceae (Al-daihan & Bhat, 2012) is a sweet edible fruit. The fruit is a drupe in which an outer fleshy part consists of pulp and a pericarp surrounding a shell of a hard endocarp with a seed inside (Farheena, Avneesh & Ugma, 2015). Date fruit comprises more than 70% sugar mainly glucose and fructose, making it a promising replacement for sugar (sucrose) in a cookie recipe, and a great nutritional benefit to diabetics and other metabolic health-related patients (Dada et al., 2012). Besides, the date fruit is rich in fiber (Hamza et al., 2014), antioxidant/flavonoids such as beta-carotene, lutein, and zeaxanthin, also minerals/vitamins like iron, calcium, copper, magnesium, potassium, and a minor source of vitamins A, and B2 (Dada et al., 2012; Farheena, Avneesh & Ugma, 2015). Pineapple belongs to the Bromeliaceae family, with fruit-equipped high-quality fiber, a source of bromelain, and a resource for a meat-tenderizing enzyme (d’Eeckenbrugge et al., 2011). Pineapple fruit remains a good source of vitamin B1, vitamin B6, including copper and dietary fiber, calcium, potassium, fiber, and vitamin C, as well as promising antioxidant/polyphenolic compounds, alongside low fat and cholesterol (Joy & Anjana, 2015; Hossain & Rahman, 2011). Processed pineapple fruit provides such products as canned slices, pulp, dried (pineapple), pasteurized juice, and concentrate. Besides fresh pineapple juices being popular given their pleasant aroma, flavor, and numerous functional properties (Rattanathanalerk, Chiewchan & Srichumpoung, 2005), it is considered a functional/nonalcoholic drink given their health-promoting properties, anti-inflammatory, antiatherosclerotic, antiaging, and many other healing properties (FAO, 2001). Pineapple juice has a proximate composition of 81.2–86.2% moisture, and 13–19% total solids, with sucrose, glucose, and fructose are the main compositions, 0.4% fibre, and a rich source of vitamin C (Dull, 2000). More so, the demand for pineapple fruit increases with the awareness of its health benefits (Nwachukwu & Ezejiaku, 2014).

Baking is among cooking/food processing methods that subjects mixed raw ingredients to prolonged heating *via* conductive/convective mode ovens, which allows heat to penetrate from product’s surface to its center, and producing distinct color, aroma, and texture. Different factors do influence the finished product quality especially during the baking process, *e.g*., temperature, time, relative humidity, and mode of heating. Among them, the baking temperature seems to carry very significance (Monteau et al., 2018). The consumption of cookies is high in Nigeria, with the major raw material being wheat flour, accounting for about 90% of the total (raw material) input. Indigenous materials raw materials like date flour produced from date fruit pulp and pineapple juice are nutrient rich, which when supplemented with wheat flour can increase the (nutrient) requirement of cookies. Also, temperature would influence baked product quality, so a study on the effects of temperature is necessary, which would mainly depend upon the principal raw ingredients. Therefore, studying the effects of temperature on cookies are necessary especially if the impact on quality attributes are to be deduced. More so, blending wheat flour, date flour, and pineapple juice could improve the nutrient quality needed in modern-day cookies. To supplement existing literature, therefore, this current study investigated the quality characteristics of date and wheat flour pineapple juice blended cookies as affected by different baking temperatures.

## Materials and Methods

### Preparation of experimental samples

**Figure 1:**
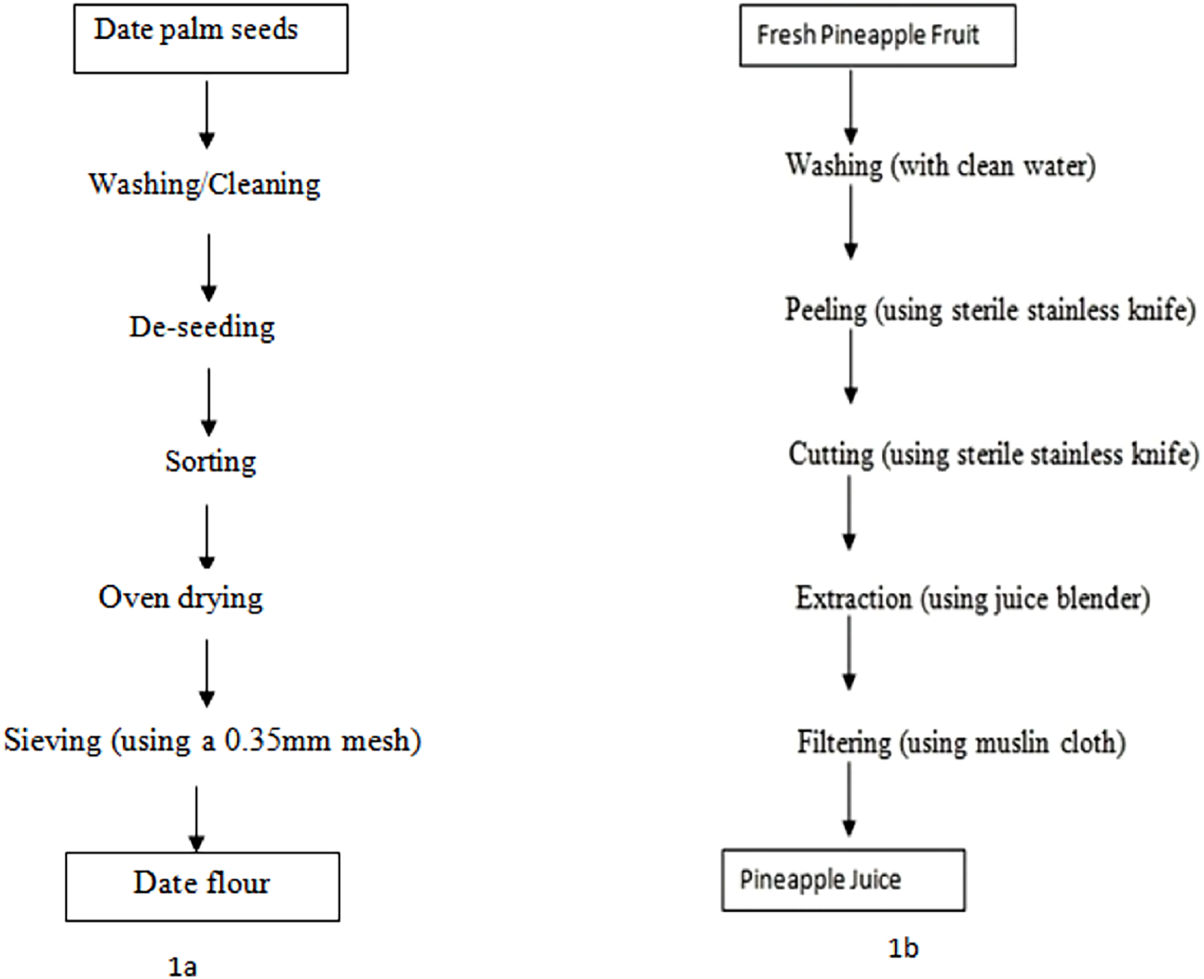
Schematic production flow of date flour (1A) and pineapple juice (1B).

Wheat flour, pineapple, baking fat, and sugar were procured from Ogige Market in Nsukka, Enugu State while date fruit was procured from Katako Market in Jos, Plateau State. The date palm fruit pulp (powder) was produced by washing the date palm fruits with water to remove adhering dirt, followed by removing the seeds (de-pitting) of the fruit manually and cut into small pieces with the aid of a knife and weighing the dried date palm fruit. The pulp with pericarp was then oven dried at 75 °C for 6–8 h and subsequently milled using a hand milling machine and sieved through a 0.35 mm mesh sieve to obtain fine homogenized particles (Peter-Ikechukwu et al., 2017). The pineapple juice was made from ripe pineapples. The pineapples were washed with clean water, peeled, and cut/diced with clean knives. The pineapple pulps were blended using a juice blender and the juice was filtered using a muslin cloth to remove all solids and colloidal particles. Schematic production flow of date flour and pineapple juice are shown in Figs. 1A and 1B, respectively.

### Formulation and production of cookies

**Figure 2:**
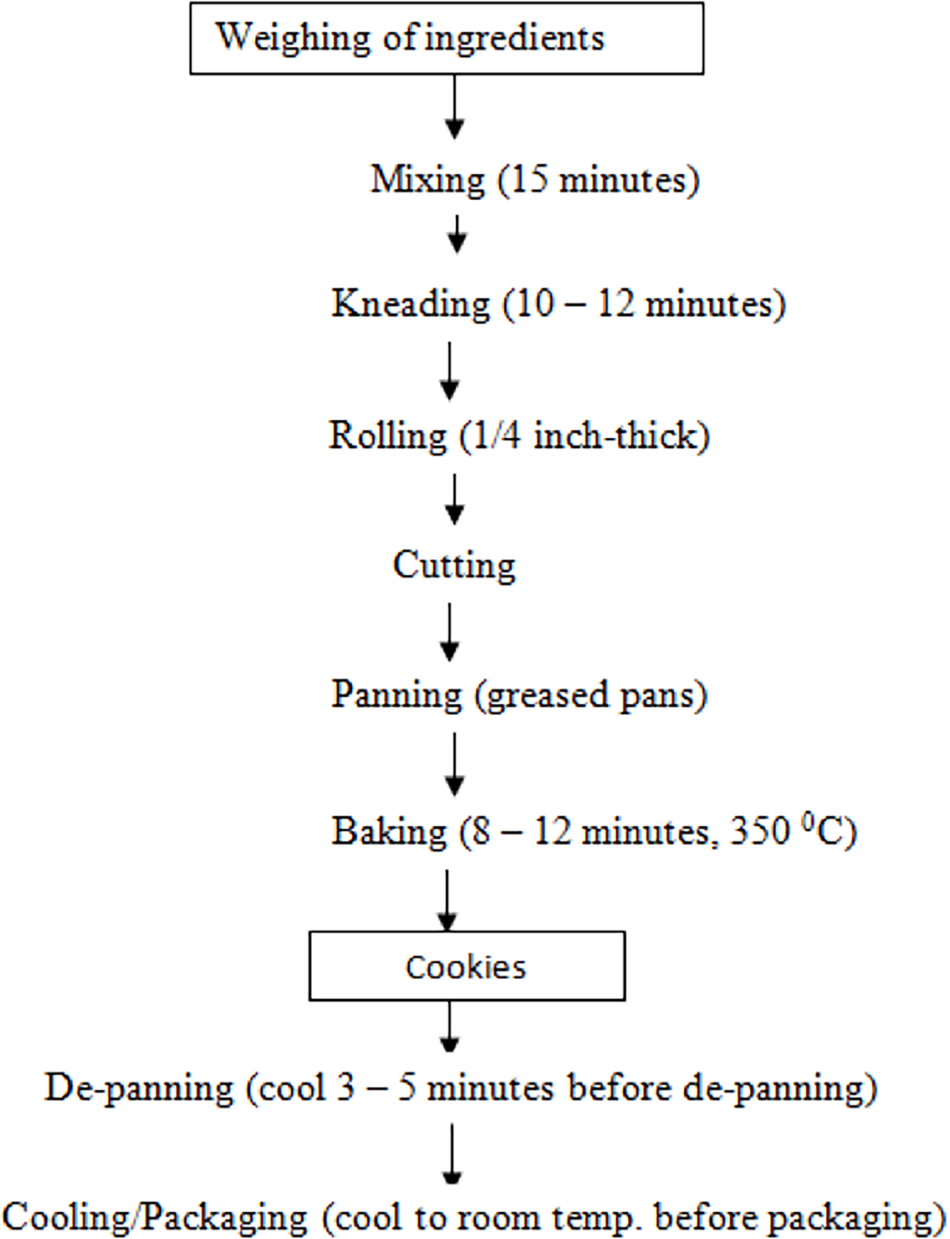
Schematic flow of cookies production.

**Figure 3:**
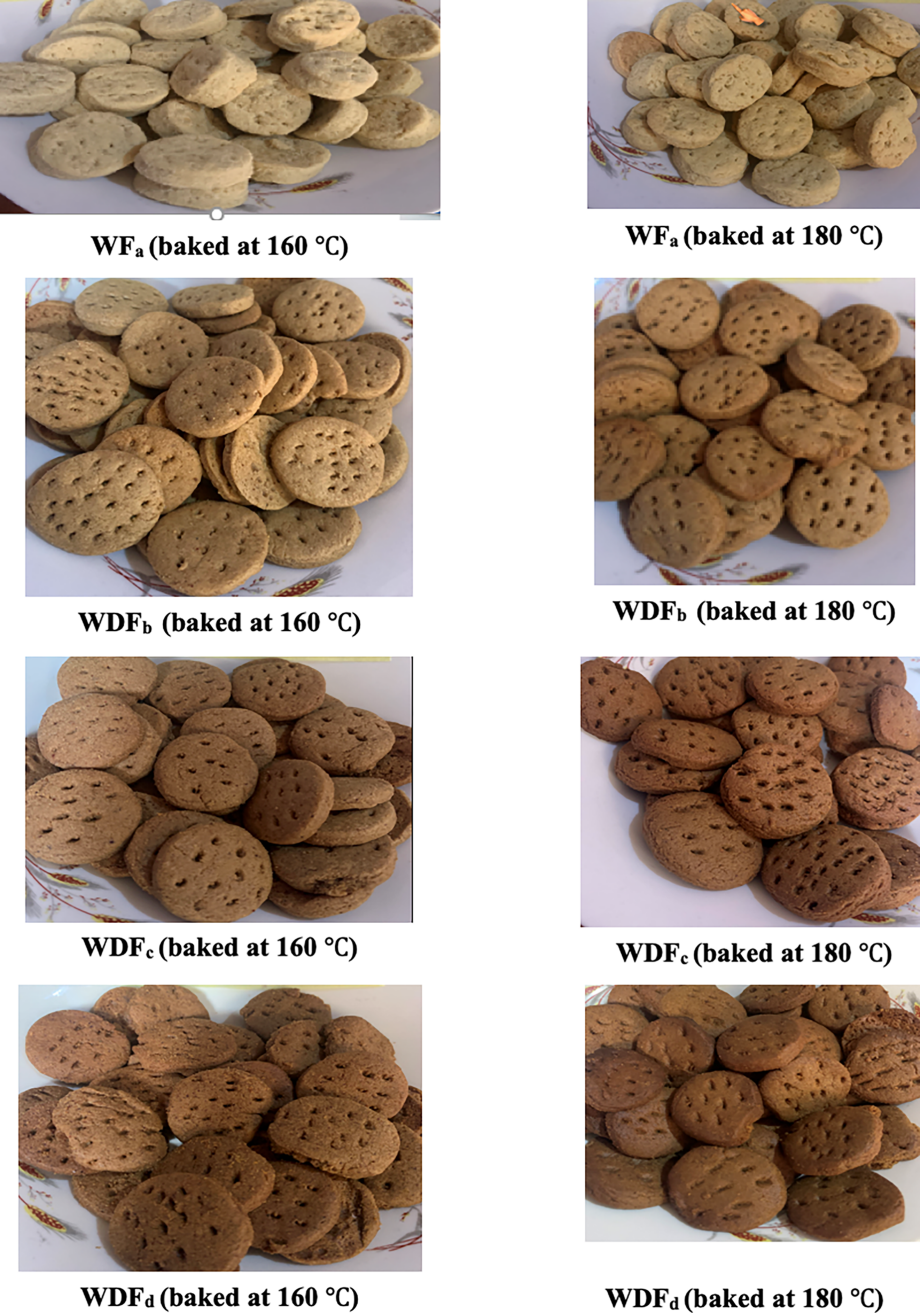
The pictorial outcomes of different cookies samples obtained from blend formulation. WF_a_ = 100% wheat flour, WDF_b_ = 90% wheat flour, 10% date flour, WDF_c_ = 80% wheat flour, 20% date flour, WDF_d_ = 70% wheat flour, 30% date flour.

A two-component augmented mixture design was used to assemble the blends of wheat and date flours by sample ratio, which were as follows: WFa (100:0) representing 100% wheat flour, 0% date flour; WDFb (90:10) representing 90% wheat flour, 10% date flour; WDFcm (80:20) representing 80% wheat flour, 20% date flour; and WDFd (70:30) representing 70% wheat flour, 30% date flour. This enabled the study design to produce four mixtures, with proportions for each ingredient applicable to each treatment combination. Additionally, the recipe for cookies production involved the following ingredients with their respective quantities: flour = 500 g, sugar = 100 g, baking fat = 180 g, salt = 2 g, baking powder = 2.5 g, baking soda = 2 g, water, = 80 mL and pineapple juice = 80 mL. The ingredients were weighed and mixed thoroughly into a dough. The dough was rolled on a board (with flour spread on it to avoid sticking) using a rolling pin. The rolled batter was cut into shapes and arranged on a greased baking tray and were placed in an electric oven facility (Century 371 OVEN COV-8320; Century Home Appliances, Nigeria). According to Sani et al. (2014), the control of baking process using an oven requires the manipulation of temperature and time operations. In this current work, the standard recipe for baking cookies involved two different temperatures of 160 °C and 180 °C, which was within those used by Sani et al. (2014). In addition, the baking time was approximately 30 min. After the baking process had completed, the prepared oven baked cookies were brought out, cooled, packaged using a hermetically sealed high density polyethylene bag with zip, and kept under ambient conditions (24–48 h) during which all the analytical measurements were performed. For emphasis, the schematic flow of cookies production is shown in Fig. 2. The pictorial outcomes of different cookies samples obtained from blend formulation is shown in Fig. 3.

### Analytical measurements of quality attributes

#### Determination of proximate analysis and energy values

Proximate compositions, which included moisture content, crude fibre, crude fat, crude ash, crude protein, and carbohydrate, together with energy values of samples were determined using the official methods of analysis (AOAC, 2019).

#### Determination of functional properties

Functional properties involved bulk density, and water and oil absorption capacities. The bulk density of the flour blends was determined according to the method of Okaka & Potter (2005). A previously weighed measuring cylinder was filled to the 10 mL mark with the sample. The bottom of the cylinder was tapped gently but repeatedly on a laboratory bench until there was no further reduction of the sample level. The cylinder with the sample was weighed.

The bulk density (BD) was calculated as:

${\rm BD \;(g/cm^3)}= {{W2-W1}\over{V}}$

where: BD = Bulk density (g/cm^3^); W_1_ = Weight of empty cylinder (g); W_2_ = Volume of cylinder + sample (g); V = Volume of cylinder occupied by the sample (cm^3^).

The water and oil absorption capacities of the formulated samples were determined using the method described by Onwuka (2018) with slight modifications. About 1 g of each of the samples was weighed into a conical graduated centrifuge tube, and then a warring whirl mixer was used to thoroughly mix the sample with 10 mL of distilled water or oil for 30 min. The mixture was allowed to stand for 30 min at room temperature and then centrifuged at 5,000×*g* for 30 min. The volume of free water or oil (supernatant) was read directly from the graduated centrifuge tube. The absorption capacity was expressed as a gram of oil or water absorbed (or retained) per gram of sample.

#### Determination of physical components

Physical components included the determinations of diameter (cm), thickness (cm), and average weight (g) of the cookies, which helped to calculate the speed ratio (SR), volume (cm^3^), and density (g/cm^3^) values according to the Approved Methods of the American Association of Cereal Chemists (American Association of Cereal Chemists, 2000).

#### Determination of minerals and vitamins

Minerals and vitamin contents, which included the determinations of iron, calcium, and potassium, were performed as described by AOAC (2019) with slight modifications. For iron and potassium, we employed the atomic absorption spectrophotometer (AAS) approach. One gram (1 g) of the sample was first digested with 30 ml of aqua regia which is a mixture of concentrated HNO3 and HCL in the ratio of 1:3. The digested sample was filtered and made up to 50 ml with deionized water. The aliquots of the digested filtrate was used for AAS using filters that match the different elements. For calcium content, one milliliter of the filtrate was pipette into duplicate tubes, then 3 ml calcium working reagent consisting of dye solution salt (0.18 g) methythymol blue, 6.0 polyvinyl pyrolidone, 7.2 g hydroxyquinoline, 10 ml hydrochloric acid concentrated, 1 l of distilled water was added and shaken for 10 min, absorbance was taken at 612 nm against a blank using a spectrophotometer.

To determine the beta-carotene, the the method described by Onwuka (2018) with slight modifications. A total of 1 g of the sample each was extracted by mixing with 20 ml of petroleum ether. The extract was evaporated to dryness and the residue dissolved with 0.2 ml chloroform-acetic anhydride mixture. A total of 2 ml of trichloro acetic acid (TCA) was also added to the extract mixed thoroughly and the absorbance read at 620 nm within 15 s. With the absorbance value, beta-carotene was calculated thus:

$\rm Concentration\; (mg) =\frac{Abs \times volume \;of\;cuvetta \times Df}{E}$

where: Abs = Absorbance, Df = Dilution factor, E = Extinction coefficient.

To determine the vitamin C content, the method described by Onwuka (2018) was used with slight modifications. One gram (1 g) of the sample and 2.5 ml of 20% meta-phosphoric acid (as stabilizing agent) was added and weighed into a 100 ml volumetric flask. Ten (10) ml of the solution was mixed with 2.5 ml of acetone and homogenized. The absorbance reading of 264 nm wavelength using ultraviolet spectrophotometer gave the vitamin C content.

${\rm Vitamin\; C}={\frac {Absorbance\; of\; test \times dilution\; factor}\over{Slops\;(from \;statndard\; curves)}}$

#### Determination of microbial load

Microbial load determinations involved total viable count (TVC) and mould count (MC). With respect to TVC, the method of Prescott, Harley & Klein (2005) was used with slight modification. One ringer tablet was dissolved in distilled water (500 mL). The clear solution formed was sterilized in the autoclave for 15 min at 12 °C. The ringer’s solution was allowed to cool completely to a temperature of about 28 °C. One gram of each sample was weighed and put in test tubes prepared for serial dilution. Ringer’s solution (9 mL) was added to all the test tubes having the samples and the mixtures were properly homogenized by shaking. A dilution of 10^−2^ was used for the total viable count determination. Then, 1 mL of the diluents was transferred into sterile disposable Petri dishes. Also, 20 mL of the sterile nutrient agar was poured into each petri dish and swirled to mix properly. The mixtures were allowed to solidify and thereafter turned upside. They were cultured by incubating at the temperature of about 37 °C for 24 h. At the end of the incubation period, colonies were counted and estimated using the colony counter and were calculated as colony-forming units per gram (cfu/g of the samples). The averages of the colonies from duplicates were determined.

${\rm TVC \;(cfu/g) = Average\; number \;of\; colonies \times reciprocal \;of\; dilution \;factor}$

With respect to mould count, method described by Prescott, Harley & Klein (2005) was used with slight modification. The preparations of the reagents followed the same steps as stated in the determination of the total viable count. The only difference was the SDA used in media preparation for mould count determination. One milliliter (1 mL) of each dilution (10^−1^) was pipetted into duplicate Petri dishes. Also, 15 mL of SDA (Sabaroud and Dextrose Agar) was pipetted aseptically into the Petri dishes. These Petri dishes were swirled in order to mix the content thoroughly, allowed to solidify and turned upside down thereafter. Petri dishes were incubated for 48 h at room temperature. The mould counts were expressed as cfu/g (colony forming unit per gram) of the samples.

${\rm Mould \;count \;(cfu/g) = Average\; number\; of \;colonies \times reciprocal\; of\; the \;dilution\; factor.}$

#### Determination of sensory attributes

The cookies samples were evaluated for color, flavour, taste after taste, texture, mouthfeel, crispness and overall acceptability on a nine-point Hedonic scale where nine (like extremely) was the highest and one (dislike extremely) was the lowest score. The evaluation was done by 20 semi-trained panelists selected from the students of the Department of Food Science and Technology, University of Nigeria, Nsukka. The panelists during their studies had undertaken sensory training, and therefore were familiar regards various evaluation criteria specific to the cookies. Participation was voluntary, verbal consent was taken, and no information regards the panelists were taken. The samples were coded and presented without further treatment in coded plastic plates to the panelists randomly during the day. The order of presentation of the samples was randomized. Clean water was presented to the panelist to rinse their mouth in between samples, to ensure the previous sample tested did not influence the new, adhering to report of Çakmakçı et al. (2015). The room where the sensory evaluation was conducted was quiet, well ventilated and free from distraction.

### Statistical analysis

Results from two determinations independently measured from different samples were submitted to analysis of variance (ANOVA). Duncan’s new multiple range tests (DNMRT) were used to compare the treatment means. Statistical significance was accepted at *p* < 0.05. Statistical Product for Service Solution (SPSS) version 25.0 was used to run the data.

## Results and discussion

### Temperature effects on cookies’ proximate composition and energy

#### Moisture content

In Table 1, the moisture content ranged from 6.89–7.40% with sample WDF_d_ (6.89%) and WDF_c_ (7.40%) having the least and highest moisture content, respectively. Significant (*p* < 0.05) differences were observed between all the samples. Increasing the baking temperature from 160–180 °C steadily decrease in moisture content, like samples WDF_b_ (7.26–6.61%)_,_ WDF_c_ (7.58–7.23%) and others. Besides samples with the same formulation baked at two temperatures produced different moisture content effects, there was significant interaction (*p* < 0.05) between the formulation and baking temperature, seemingly to agree with data of Prescott (2004). Low moisture content values were important in the formulation since high moisture levels would accelerate spoilage during storage.

**Table 1:** Proximate composition and energy values of cookies produced from blends of wheat and date flour with pineapple juice.

Sample	Temperature (°C)	Moisture (%)	Proteins (%)	Fat (%)	Fibre (%)	Ash (%)	Carbohydrate (%)	Energy (kcal)
WF_a_	160	7.85 ± 0.01	10.19 ± 0.01	15.94 ± 0.02	1.01 ± 0.01	0.64 ± .05	64.39 ± 0.01	441.74 ± 0.30
	180	6.48 ± 0.04	10.24 ± 0.01	16.04 ± 0.02	1.04 ± 0.01	0.91 ± 0.01	65.30 ± 0.06	446.48 ± 0.09
	Total	7.16^c^ ± 0.79	10.22^d^ ± 0.03	15.99^d^ ± 0.06	1.02^a^ ± 0.02	0.77^a^ ± 0.16	64.85^a^ ± 0.53	444.10^c^ ± 2.74
WDF_b_	160	7.26 ± 0.02	9.53 ± 0.02	15.34 ± 0.02	1.03 ± 0.01	0.60 ± 0.01	66.27 ± 0.02	441.18 ± 0.19
	180	6.61 ± 0.02	9.62 ± 0.01	15.45 ± 0.02	1.09 ± .014	1.22 ± 0.03	66.03 ± 0.02	441.57 ± 0.08
	Total	6.93^b^ ± 0.38	9.57^c^ ± 0.05	15.39^c^ ± 0.07	1.06^b^ ± 0.04	0.91^b^ ± 0.36	66.15^b^ ± 0.14	441.37^c^ ± 0.25
WDF_c_	160	7.58 ± 0.01	9.37 ± 0.03	14.43 ± 0.01	1.08 ± 0.02	0.52 ± 0.03	67.03 ± 0.01	435.47 ± 0.07
	180	7.23 ± 0.04	9.46 ± 0.01	14.55 ± 0.02	1.07 ± 0.02	1.31 ± 0.01	66.40 ± 0.08	434.33 ± 0.18
	Total	7.40^d^ ± 0.20	9.42^b^ ± 0.06	14.49^b^ ± 0.07	1.07^b^ ± 0.02	0.92^b^ ± 0.46	66.71^c^ ± 0.37	434.90^b^ ± 0.67
WDF_d_	160	7.42 ± 0.03	8.70 ± 0.02	14.36 ± 0.01	1.10 ± 0.00	0.80 ± 0.01	67.63 ± 0.04	434.50 ± 0.02
	180	6.35 ± 0.04	8.77 ± 0.02	14.39 ± 0.01	1.12 ± 0.01	1.60 ± 0.01	67.80 ± 0.04	435.71 ± 0.16
	Total	6.89^a^ ± 0.62	8.73^a^ ± 0.04	14.37^a^ ± 0.02	1.11^c^ ± 0.01	1.20^c^ ± 0.45	67.71^d^ ± 0.10	435.10^a^ ± 0.70
Total	160	7.52 ± 0.23	9.45 ± 0.57	15.01 ± 0.70	1.05 ± 0.04	0.64 ± 0.11	66.33 ± 1.30	438.22 ± 3.49
	180	6.66 ± 0.36	9.52 ± 0.56	15.10 ± 0.72	1.08 ± 0.03	1.26 ± 0.26	66.38 ± 0.97	439.52 ± 5.19
	Total	7.09 ± 0.53	9.48 ± 0.55	15.06 ± 0.69	1.06 ± 0.04	0.95 ± 0.38	66.35 ± 1.11	438.87 ± 4.32

**Note:**

Values are presented in means ± standard deviation of three determinations. Mean values on the same column with different superscripts are significantly (*p* < 0.05) different. WF_a_ = 100% wheat flour, WDF_b_ = 90% wheat flour, 10% date flour, WDF_c_ = 80% wheat flour, 20% date flour, WDF_d_ = 70% wheat flour, 30% date flour.

#### Protein and fat content

In Table 1, the protein content ranged from 8.73–10.22%, like with sample WF_a_ (10.22%) with 100% wheat flour as peak, and sample WDF_d_ (8.73%) with 70% wheat and 30% date flour as least protein content. An increased substitution of wheat with date flour in the formulation led decreased protein content in the cookies. According to Abdel et al. (2012), date palm fruit has a lower protein content (2.3–5.6%). Also, an increase in baking temperature led to an increase in protein content, like in samples WDF_b_ (9.57%)_,_ WDF_c_ (9.42%) and others. This steady increase could be that at high temperatures, proteins are denatured and anti-nutrients would be bound to proteins being liberated from the sample leading to available proteins at high temperatures (Fabbri & Crosby, 2015).

Significant differences (*p* < 0.05) in protein occurred across all the samples, like with sample WF_a_ with 100% wheat flour. Also, a significant (*p* < 0.05) interaction term between the formulation and the baking temperature seemed apparent. More so, the quality and nutritional content of food are a simultaneous function of both formulation and processing methods (Myers, Montgomery & Anderson-Cook, 2009). Table 1 show the fat content of the cookies ranged from 14.37–15.99% for sample WF_a_ (15.99%) with 100% wheat flour having the highest and sample WDF_d_ (14.37%) with 70% wheat and 30% date flour having the lowest fat content. Probably, fat content reduction across the formulations occurred because of date flour. Baking temperatures increased with fat content for samples with resembling formulation baked at different temperatures as seen in samples WDF_b_ (15.34– 15.45%)_,_ WDF_c_ (14.43–14.55%) and in other samples. Moreover, the lowering of moisture resulted to fat concentration in the formulated cookies. Notably, fats can withstand high-temperature processing with little changes (Xu et al., 2017). Additionally, a significant (*p* < 0.05) cross-interaction seemed apparent between the formulation and baking temperature.

#### Crude fibre and ash content

In Table 1, the values for the crude fibre content ranged from 1.02–1.11%, with sample WF_a_ (1.02%) as a minimum, and WDF_d_ (1.11%) as the maximum. The inclusion of date flour during formulation steadily increased with fibre content, similarly reported by Peter-Ikechukwu et al. (2017). Fibre may lower the blood cholesterol level and slow down the absorption of glucose. Fibre also ensures smooth bowel movements, increases satiety, and facilitates weight management. The addition of date palm pulp in flour is of nutritional importance, especially to diabetic patients (Hamza et al., 2014). The ash contents of cookies increased with an increase in the quantity of date flour which is attributable to the higher content of fibre than wheat flour. Between the formulation and temperature, a significant interaction was also observed, although there was no significant (*p* > 0.05) difference between sample WDF_b_ (1.06%) and WDF_c_ (1.07%). Besides, the ash content ranged from 0.77–1.20% with sample WF_a_ (0.77%) and WDF_d_ (1.20%) having the lowest and highest, respectively (Table 1). The addition of date flour in the formulation led to a steady increase in the ash content of the cookies as seen in samples WDF_b_ (0.60–1.22%)_,_ WDF_c_ (0.52–1.31%) and in other samples. Date palm fruits according to Abdel et al. (2012) have appreciably high mineral content. Baking temperatures increased with the ash content of the cookies, attributable to concentration effects due to loss of moisture. Between the formulation and temperature, a significant (*p* < 0.05) interaction was also observed, although there was no significant (*p* > 0.05) difference between sample WDF_b_ (0.91%) and WDF_c_ (0.92%).

#### Carbohydrate content and energy values

In Table 1, the carbohydrate content ranged from 64.85–67.71% with sample WF_a_ (64.85%) as minimum and WDF_d_ (67.71%) as maximum. Significant differences (*p* < 0.05) were observed between all the samples. The substitution of date flour in the flour blends increased with the carbohydrate content of the cookies. The results obtained in carbohydrate content were similar to the results of Peter-Ikechukwu et al. (2017). Temperature from 160–180 °C increased with carbohydrate content, attributable to the effect of the concentration due to loss of moisture at higher baking temperatures. Also presented in Table 1, the energy values of the cookies ranged from 434.90– 444.10 kcal with sample WF_a_ (444.10 kcal) as the maximum, and sample WDF_c_ (435.10 kcal) as a minimum. Between the formulation and temperature, there was no significant (*p* > 0.05) difference between sample WF_a_ (444.10 kcal) and WDF_b._ (441.37 kcal). The energy value reduction could be due to higher protein and fat contents in wheat flour (control) above those used in formulations supplemented with date flour. Also, the concentration of fats, carbohydrates, and proteins could be a result of moisture loss during formulation at high temperatures (160–180 °C) with an increase in energy content.

### Temperature effects on cookies’ minerals and vitamins

#### Calcium

The calcium content is presented in Table 2. The values ranged from 33.18–62.45 mg/100 g for sample WF_a_ (33.18 mg/100 g) with 100% wheat flour and sample WDF_d_ (62.45 mg/100 g) with 30% substitution of date flour respectively. An increased inclusion of date flour in the formulation resulted in an increase in the Ca content of the cookies with significant (*p* < 0.05) differences observed between all the samples as seen in samples WDF_b_ (53.32 mg/100 g)_,_ WDF_c_ (58.62 mg/100 g) and in other samples. Compared to wheat, the Ca content increased with inclusion of date fruit flour. This happened probably due to high amount of calcium present in date fruit (Assirey, 2015). A change in baking temperature did not significantly affect the calcium content cookies. The values obtained were in agreement with the values reported by Man et al. (2021). The values were lower than the US RDA of 700 mg/day for children and 1,000 mg/100 g for adults (WebMD, 2021).

**Table 2:** Micronutrient content of cookies produced from blends of wheat and date flour with pineapple juice.

Sample	Temperature (°C)	Calcium (mg/100 g)	Iron (mg/100 g)	Potassium (mg/100 g)	Vitamin A (mg/100 g)	Vitamin C (mg/100 g)
WF_a_	160	32.68 ± 0.01	3.47 ± 0.02	100.07 ± 0.03	2.01 ± 0.02	0.04 ± 0.01
	180	33.68 ± 0.01	3.48 ± 0.02	100.07 ± 0.03	1.97 ± 0.01	0.03 ± 0.01
	Total	33.18^a^ ± 0.58	3.47^a^ ± 0.02	100.07^a^ ± 0.02	1.99^a^ ± 0.03	0.04^a^ ± 0.01
WDF_b_	160	53.31 ± 0.00	4.26 ± 0.02	312.34 ± 0.01	4.07 ± 0.00	0.09 ± 0.01
	180	53.32 ± 0.00	4.27 ± 0.03	312.34 ± 0.01	4.06 ± 0.02	0.07 ± 0.00
	Total	53.32^b^ ± 0.01	4.26^b^ ± 0.02	312.34^b^ ± 0.01	4.06^b^ ± 0.01	0.08^b^ ± 0.01
WDF_c_	160	58.62 ± 0.02	5.37 ± 0.01	321.74 ± 0.03	4.47 ± 0.02	0.11 ± 0.00
	180	58.62 ± 0.03	5.38 ± 0.01	321.73 ± 0.03	4.35 ± 0.01	0.10 ± 0.00
	Total	58.62^c^ ± 0.02	5.38^c^ ± 0.01	321.74^c^ ± 0.02	4.41^c^ ± 0.07	0.12^c^ ± 0.01
WDF_d_	160	62.45 ± 0.01	5.75 ± 0.01	358.63 ± 0.02	4.91 ± 0.00	0.17 ± 0.01
	180	62.46 ± 0.01	5.75 ± 0.01	358.63 ± 0.01	4.87 ± 0.02	0.14 ± 0.01
	Total	62.45^d^ ± 0.01	5.75^d^ ± 0.01	358.63^d^ ± 0.02	4.89^d^ ± 0.02	0.15^d^ ± 0.02
Total	160	51.76 ± 2.28	4.71 ± 0.97	273.20 ± 8.44	3.86 ± 1.19	0.10 ± 0.05
	180	52.01 ± 1.84	4.72 ± 0.96	273.19 ± 1.44	3.81 ± 1.18	0.08 ± 0.04
	Total	51.89 ± 1.65	4.71 ± 0.93	273.19 ± 4.77	3.84 ± 1.14	0.09 ± 0.04

**Note:**

Values are presented in means ± standard deviation of three determinations. Mean values on the same column with different superscripts are significantly (*p* < 0.05) different. WF_a_ = 100% wheat flour, WDF_b_ = 90% wheat flour, 10% date flour, WDF_c_ = 80% wheat flour, 20% date flour, WDF_d_ = 70% wheat flour, 30% date flour.

#### Iron

In Table 2, the iron content of the cookies ranged from 3.47–5.75 mg/100 g for sample WF_a_ (3.47 mg/100 g) and sample WDF_d_ (5.75 mg/100 g), respectively. The iron content increased in the formulation with the increased inclusion of date flour as seen in samples WDF_b_ (2.26 mg/100 g)_,_ WDF_c_ (5.38 mg/100 g) and in other samples. This could be due to the high iron content of date flour compared to wheat flour. Significant (*p* < 0.05) differences were observed in the iron content with sample WDF_d_ (5.75 mg/100 g) with 30% substitution significantly higher than the other samples. The two baking temperatures employed in the production did not significantly affect (*p* > 0.05) the iron content of the cookies. The results obtained for the iron content were higher than the values (1.40–2.76 mg/100 g) reported by Man et al. (2021).

#### Potassium

In Table 2, the potassium content of the cookies ranged from 100.07–358.63 mg/100 g for sample WF_a_ (100.07 mg/100 g) and sample WDF_d_ (358.63 mg/100 g), respectively. An increased inclusion of date flour in the formulation resulted in an increase in the potassium content of the cookies with significant (*p* < 0.05) differences observed between all the samples as seen in samples WDF_b_ (312.34 mg/100 g)_,_ WDF_c_ (321.74 mg/100 g) and in other samples. An increase in the potassium content with increased inclusion of date flour could be due to the high amount of potassium reported to be present in date flour compared to wheat. A change in baking temperature did not significantly affect the potassium content of the cookies.

#### Vitamin A and C

In Table 2, the vitamins A and C contents increased with the increased addition of date flour in the formulation. Sample WFa (1.99, 0.04 mg/100 g) with 100% wheat flour was significantly lower than all the others in terms of both vitamin A and C and sample WDF_d_ (4.89, 0.15 mg/100 g) with 30% substitution of date flour was higher than the other samples. This could be due to the high amount of vitamins A and C concentrated in date flour. The vitamins A and C contents were higher for samples with the same formulation and baked at 160 °C compared to samples baked at 180 °C. Vitamins A and C are sensitive to temperature changes and degrade at high temperatures. This could explain the reason why an increase in baking temperature reduced the vitamin A and C contents of the cookies as seen in samples WDF_b_ (4.07–4.06 mg/100 g)_,_ WDF_c_ (4.47–4.35 mg/100 g) and in other samples. Also, a significant interaction between the formulation and baking temperature suggests these vitamins in cookies simultaneously function with the formulation/treatment given to the sample (Myers, Montgomery & Anderson-Cook, 2009). The values obtained were lower than US RDA values for vitamin A (700–900 µg/100 g) and vitamin C (40–120 mg/100 g).

### Temperature effects on cookies’ physical properties

#### Average mass

The average mass of the cookies ranged from 7.49–7.75 g for samples WDF_d_ (7.49 g) and WF_a_ (7.75 g), respectively (Table 3). No significant (*p* > 0.05) differences were observed between samples WDF_c_ (7.49 g) and WDF_d_ (7.49 g) with 20% and 30% substitution of wheat flour with date flour. Sample WF_a_ (7.75 g) with 100% wheat flour was significantly (*p* < 0.05) higher than all the samples in terms of average mass. The high average mass could be due to the fact that nutrients and matter are more concentrated in wheat flour compared to date flour. Results for average mass were lower than the values of Man et al. (2021) for biscuits produced from roasted flaxseed flour partially substituted with wheat flour.

**Table 3:** Physical properties of cookies produced from blends of wheat and date flour with pineapple juice.

Sample	Temperature (°C)	Average mass (g)	Diameter (cm)	Thickness (cm)	Volume (cm^3^)	Density (g/cm^3^)	Spread ratio
WFa	160	7.75 ± 0.01	3.50 ± 0.00	1.20 ± 0.00	3.77 ± 0.00	2.06 ± 0.00	2.92 ± 0.00
	180	7.74 ± 0.01	3.50 ± 0.00	1.20 ± 0.00	3.77 ± 0.00	2.06 ± 0.00	2.92 ± 0.00
	Total	7.75^c^ ± 0.01	3.50^a^ ± 0.00	1.20^c^ ± 0.00	3.77^c^ ± 0.00	2.06^a^ ± 0.00	2.92^a^ ± 0.00
WDF_b_	160	7.50 ± 0.00	3.80 ± 0.00	1.12 ± 0.02	3.50 ± 0.07	2.14 ± 0.04	3.40 ± 0.06
	180	7.51 ± 0.01	3.80 ± 0.01	1.11 ± 0.02	3.49 ± 0.06	2.15 ± 0.03	3.41 ± 0.06
	Total	7.50^b^ ± 0.01	3.80^b^ ± 0.01	1.11^b^ ± 0.02	3.50^b^ ± 0.06	2.15^b^ ± 0.04	3.41^b^ ± 0.06
WDF_c_	160	7.49 ± 0.00	3.97 ± 0.04	0.99 ± 0.01	3.11 ± 0.02	2.41 ± 0.02	4.01 ± 0.02
	180	7.49 ± 0.00	4.00 ± 0.03	0.99 ± 0.01	3.11 ± 0.03	2.40 ± 0.02	4.03 ± 0.02
	Total	7.49^a^ ± 0.00	3.98^c^ ± 0.04	0.99^a^ ± 0.01	3.11^a^ ± 0.02	2.41^c^ ± 0.02	4.02^c^ ± 0.0
WDF_d_	160	7.49 ± 0.00	4.03 ± 0.02	0.99 ± 0.01	3.12 ± 0.02	2.40 ± 0.01	4.05 ± 0.04
	180	7.49 ± 0.00	3.99 ± 0.02	0.99 ± 0.01	3.10 ± 0.03	2.42 ± 0.02	4.05 ± 0.04
	Total	7.49^a^ ± 0.00	4.01^d^ ± 0.03	0.99^a^ ± 0.01	3.11^a^ ± 0.03	2.41^c^ ± 0.02	4.05^c^ ± 0.03
Total	160	7.56 ± 0.12	3.82 ± 0.21	1.08 ± 0.09	3.38 ± 0.29	2.25 ± 0.16	3.60 ± 0.48
	180	7.56 ± 0.11	3.82 ± 0.21	1.07 ± 0.09	3.37 ± 0.29	2.26 ± 0.16	3.60 ± 0.48
	Total	7.56 ± 0.11	3.82 ± 0.21	1.07 ± 0.09	3.37 ± 0.28	2.25 ± 0.16	3.60 ± 0.48

**Note:**

Values are presented in means ± standard deviation of three determinations. Mean values on the same column with different superscripts are significantly (*p* < 0.05) different. WF_a_ = 100% wheat flour, WDF_b_ = 90% wheat flour, 10% date flour, WDF_c_ = 80% wheat flour, 20% date flour, WDF_d_ = 70% wheat flour, 30% date flour.

#### Diameter

The diameter of the cookies ranged from 3.50–4.01 cm for the sample WF_a_ (3.50 cm) with 100% wheat flour and sample WDF_d_ (4.01 cm) substituted with 30% date flour, respectively (Table 3). An increase in the substitution of date flour in the formulation led to an increase in the diameter of the samples with significant (*p* < 0.05) differences as seen in samples WDF_b_ (3.80 cm)_,_ WDF_c_ (3.98 cm) and in other samples. The diameter increased with inclusion of date flour, probably because of reduced protein gluten. Moreover, substituting wheat decreased dough viscosity and resulted in a high flow rate leading to increased diameter of the cookies (Ho & Abdul-Latif, 2016). Values obtained for average diameter were lower than values earlier reported by Dhankhar, Vashistha & Sharma (2019) for biscuits produced from refined wheat flour and substituted with chickpea flour and date powder.

#### Thickness

The thickness of the cookies ranged from 0.99–1.20 cm for samples WDF_d_ (0.99 cm) and WF_a_ (1.20 cm), respectively (Table 3). Increased substitution of date flour in the formulation resulted in a decrease in the thickness as seen in samples WDF_b_ (1.11 cm)_,_ WDF_c_ (0.99 cm) and in other samples. For samples with the same formulation and baked at different temperatures, there was no significant (*p* > 0.05) difference in thickness. Such a decrease in the thickness could be due to the dilution of wheat gluten by date fruit (Aslam et al., 2014). The high water absorption characteristic of date insoluble fibre can attract more water and decrease the dough’s viscosity leading to decreased thickness (Jamilah et al., 2011). Results obtained in the thickness of samples were lower than thickness values reported elsewhere (Ho & Abdul-Latif, 2016; Peter-Ikechukwu et al., 2020).

#### Volume

The volume of the cookies ranged from 3.11–3.77 cm^3^ for sample WDF_d_ (3.11 cm^3^) with 30% date flour and sample WF_a_ (3.77 cm^3^) with 100% wheat flour, respectively (Table 3). An increased inclusion of date flour in the formulation led to a decrease in the volume with significant (*p* < 0.05) differences observed among the samples except for samples WDF_c_ (3.11 cm^3^) and WDF_d_ (3.11 cm^3^). Samples with the same formulation despite being baked at different temperatures show no significant (*p* > 0.05) difference by volume. Reduction in volume with increased substitution of wheat flour with date flour could associate with the high fibre content of date fruit flour. Fiber can help reduce dough spread during baking, which strengthens its resistance to deformation (Blanco Canalis et al., 2017). However, the resistance to deformation could occur because of high fiber content in the date flour.

#### Density

The density of the cookies ranged from 2.06–2.41 g/cm^3^ for sample WF_a_ (2.06 g/cm^3^) with 100% wheat flour and sample WDF_d_ (2.41 g/cm^3^) with 30% date flour, respectively (Table 3). Results for the density of cookies showed that an increase in the quantity of date flour in the formulation increased the density of the cookies as seen in samples WDF_b_ (2.14–2.15 g/m^3^)_,_ WDF_c_ (2.40–2.41 g/cm^3^) and in other samples. Sample WDF_d_ (2.41 g/cm^3^) with 30% substitution was significantly (*p* < 0.05) higher than the other samples. No significant (*p* > 0.05) difference happened between samples with resembling formulation and baked at the two baking temperatures (160 and 180 °C). Probably, the increase in density of the cookies could arise from the addition of fibrous matter coming from date fruit flour.

#### Spread ratio

The spread ratio of the cookies ranged from 2.92–4.05 for sample WF_a_ (2.92) with 100% wheat flour and sample WDF_d_ (4.05) with 30% date flour, respectively (Table 3). The result showed that an increased inclusion of date flour in the cookies increased the spread ratio with sample WDF_d_ (4.05) significantly (*p* < 0.05) higher than the other samples. The increase in spread ratio with the increased addition of date flour could be due to the restriction of dough viscosity. Cookie spread represents the ratio of diameter to thickness. Cookies’ spread ratio appears to be controlled by dough viscosity; dough with low viscosity causes cookies to spread more rapidly compared those with high viscosity (Ho & Abdul-Latif, 2016). Cookies with higher spread ratio should be most desirable compared those with low spread ratio (Handa, Goomer & Siddhu, 2012). Cookies results herein obtained in the spread ratio seemed to agree with data reported by Peter-Ikechukwu et al. (2020) about cookies produced from date fruit pulp, toasted watermelon seed, and wheat flour composite.

### Temperature effects on cookies’ functional properties

#### Water absorption capacity

In Table 4, the water absorption capacity (WAC) of the flour samples ranged from 1.14–1.18 g/g for sample WF_a_ (1.18 g/g) with 100% wheat flour and sample WDF_d_ (1.14 g/g) with 30% date fruit flour substitution, respectively. Increased addition of date flour in the formulation reduced the WAC with sample WF_a_ significantly (*p* < 0.05) higher than the other samples. High WAC in sample WF_a_ (1.18 g/g) could be due to protein and starch molecules in wheat flour creating hydrogen bonds and hydrophilic interactions with the added water molecules in the flour. Water molecules hydrate the gluten-forming proteins gliadin and glutenin, as well as damaged starch and other ingredients (BAKERpedia, 2021). Lower values of WAC in samples WDF_b_ (1.15 g/g), WDF_c_ (1.14 g/g), and WDF_d_ (1.14 g/g) could therefore be due to a dilution effect of wheat starch and protein by date flour. Values obtained for water absorption capacity were in the range of values (1.00 to 1.60) reported by Oladele et al. (2017).

**Table 4:** Functional properties of wheat and date flour.

Sample	WAC (g/g)	OAC (g/g)	BD (g/ml)
WF_a_	1.18^c^ ± 0.00	1.31^a^ ± 0.00	0.76^b^ ± 0.00
WDF_b_	1.15^b^ ± 0.00	1.32^b^ ± 0.00	0.74^a^ ± 0.00
WDF_c_	1.14^a^ ± 0.00	1.33^c^ ± 0.00	0.74^a^ ± 0.00
WDF_d_	1.14^a^ ± 0.00	1.33^c^ ± 0.01	0.74^a^ ± 0.00

**Note:**

Values are presented in means ± standard deviation of three determinations. Mean values on the same column with different superscripts are significantly (*p* < 0.05) different. WF_a_ = 100% wheat flour, WDF_b_ = 90% wheat flour, 10% date, WDF_c_ = 80% wheat flour, 20% date flour, WDF_d_ = 70% wheat flour, 30% date flour.

#### Oil absorption capacity

In Table 4, the oil absorption capacity (OAC) of the flour samples ranged from 1.31–1.33 g/g for sample WF_a_ (1.31 g/g) with 100% wheat flour and sample WDF_d_ (1.33 g/g) with 30% date fruit flour substitution, respectively. The oil absorption capacity as observed increased with an increased substitution of wheat flour with date flour. Fat content of the samples could also be attributed to the margarine added. Sample WF_a_ (1.31 g/g) was significantly (*p* < 0.05) lower than the other samples, with no significant (*p* > 0.05) difference seen between sample WDF_c_ (1.33 g/g) and WDF_d_ (1.33 g/g). Oil absorption in starch relies predominantly on the physical entrapment of oil within the starch structures. This is because starch does not have nonpolar sites, compared to those found in proteins (Oladele et al., 2017). The inclusion of date flour could be reducing the nonpolar site found in wheat proteins and make the formulation more polar resulting in an increased oil absorption capacity.

#### Bulk density

The bulk density of the flour samples ranged from 0.74–0.76 g/mL for sample WDF_d_ (0.74 g/mL) with 30% date fruit flour substitution and sample WF_a_ (0.76 g/mL) with 100% wheat flour and respectively (Table 4). The result showed that an increased substitution of wheat flour with date flour resulted in a decrease in the bulk density of the flour. Sample WF_a_ (0.76 g/mL) was significantly (*p* < 0.05) higher than all the other samples but no significant (*p* > 0.05) difference was observed for the different substitution levels. Bulk density is the mass per unit volume of food. It is also indicative of how porous the food product is, and how it can be packed together. Values obtained for bulk density were within the range of values earlier reported by Oladele et al. (2017) and values (0.762–0.820) reported by Chandra, Singh & Kumari (2015) for different composite flours used in the production of biscuits.

### Temperature effects on cookies’ microbial load

In Table 5, the results showed that the total viable count decreased with an increase in baking temperature. Samples with the same formulation that was baked at 160 °C were having higher counts compared to samples that were baked at 180 °C. The total viable count estimates the total number of microorganisms, such as bacteria, yeast, or mold species, that are present in a food sample and indicate the level of hygiene and how well the product was handled after processing. Results obtained were lower than the values (3.8 × 10^4^ to 7.7 × 10^4^ CFU/g) reported by Mbaeyi-Nwaoha & Uchendu (2016) for a cereal-based product from acha and fermented soybean paste. Also presented in Table 5, the results showed that mould growth was not detected in any of the formulated cookies, which suggests effectiveness of the two baking temperatures able to deactivate moulds and their spores. Ferone et al. (2020) identified microbial levels that are acceptable especially for ready-to-eat products. For instance, the total viable count (TVC)—which reports the sum of yeasts, molds, and bacteria—was considered acceptable at levels <10.000 CFU/g, borderline at levels between 10.000–≤1.000.000 CFU/g, unsatisfactory at levels >1.000.000 CFU/g. Moreover, mould growth would lead to quality loss in foods, characterised by bad smell and bad flavours, discoloration, *etc*.

**Table 5:** Microbial load of cookies produced from blends of wheat and date flour with pineapple juice.

SAMPLES	Temperature (°C)	TVC (cfu/g)	Mould Count (cfu/g)
WF_a_	160	2.9 × 10^3^	ND
WF_a_	180	2.0 × 10^3^	ND
WDF_b_	160	2.6 × 10^3^	ND
WDF_b_	180	1.7 × 10^3^	ND
WDF_c_	160	2.1 × 10^3^	ND
WDF_c_	180	1.9 × 10^3^	ND
WDF_d_	160	1.4 × 10^3^	ND
WDF_d_	180	1.1 × 10^3^	ND

**Note:**

WF_a_ = 100% wheat flour, WDF_b_ = 90% wheat flour, 10% date, WDF_c_ = 80% wheat flour, 20% date flour, WDF_d_ = 70% wheat flour, 30% date flour. ND, Not Detected.

### Temperature effects on cookies’ sensory scores

#### Colour

In Table 6, there were colour scores for cookies from blends of wheat and date flour with pineapple juice. The values ranged from 6.23–7.45 for sample WDF_b_ (6.23) and WDF_d_ (7.45), respectively. No significant (*p* > 0.05) difference was observed across samples WDF_d_ (7.45), WDF_c_ (7.10), and WDF_a_ (6.95), despite the highly rated colour for WDF_d_, probably due to the desirable brown date flour colour in the formulation. Generally, samples baked at 160 °C appeared more appealing compared to samples baked at 180 °C for the sample formulation. Baking temperatures as well as formulation significantly (*p* < 0.05) affected the colour of the blend samples. Besides Myers, Montgomery & Anderson-Cook (2009) reported that food quality depends on the combination of the mixture, together with process used in the production, Patel et al. (2019) understood that loss of desirability in colour and other sensory characteristics for de-oiled bottle gourd (*Lagenaria siceraria*) seed cake fortified biscuit occurred with increased baking temperature(s). Lower values could be due to the intense brown colour of the cookies that resulted from a caramelization reaction. The color results of cookies herein seems to agree data reported by Peter-Ikechukwu et al. (2017) for cookies produced from whole wheat and date palm fruit pulp.

**Table 6:** Sensory scores for cookies produced from blends of wheat and date flour with pineapple juice.

Sample	Temperature (°C)	Colour	Flavour	Taste	Aftertaste	Texture	Mouthfeel	Crunchiness	OA
WF_a_	160	7.70 ± 1.26	7.60 ± 0.88	7.70 ± 0.98	7.40 ± 0.99	7.10 ± 1.07	7.40 ± 1.14	6.75 ± 1.65	7.45 ± 1.10
	180	6.20 ± 1.67	6.45 ± 1.67	6.15 ± 1.76	6.20 ± 1.77	6.80 ± 1.36	6.45 ± 1.57	6.95 ± 1.39	6.55 ± 1.50
	Total	6.95^b^ ± 1.65	7.03^a^ ± 1.44	6.93^a^ ± 1.61	6.80^a^ ± 1.54	6.95^a^ ± 1.22	6.93^a^ ± 1.44	6.85^a^ ± 1.51	7.00^a^ ± 1.38
WDF_b_	160	6.35 ± 0.93	6.80 ± 1.15	6.75 ± 1.29	6.80 ± 1.20	7.20 ± 0.70	7.10 ± 1.07	7.70 ± 0.92	7.35 ± 0.81
	180	6.10 ± 1.41	6.85 ± 1.31	6.95 ± 1.23	6.45 ± 1.32	7.30 ± 0.98	7.20 ± 0.89	7.55 ± 1.36	7.25 ± 1.02
	Total	6.23^a^ ± 1.19	6.83^a^ ± 1.22	6.85^a^ ± 1.25	6.63^a^ ± 1.25	7.25^a^ ± 0.84	7.15^a^ ± 0.98	7.63^b^ ± 1.15	7.30^a^ ± 0.91
WDF_c_	160	7.60 ± 1.10	7.40 ± 1.14	7.90 ± 0.78	7.20 ± 1.36	7.40 ± 1.43	7.30 ± 1.13	7.20 ± 1.70	7.70 ± 0.92
	180	6.60 ± 0.82	6.30 ± 1.34	6.70 ± 0.92	6.50 ± 1.19	7.10 ± 1.07	6.90 ± 0.91	7.60 ± 1.19	6.85 ± 1.00
	Total	7.10^b^ ± 1.08	6.85^a^ ± 1.35	7.30^a^ ± 1.04	6.85^a^ ± 1.31	7.25^a^ ± 1.26	7.10^a^ ± 1.03	7.40^ab^ ± 1.46	7.28^a^ ± 1.04
WDF_d_	160	7.45 ± 1.10	6.90 ± 1.59	7.25 ± 1.52	6.95 ± 1.79	7.65 ± 0.81	7.40 ± 1.10	7.95 ± 1.00	7.25 ± 1.48
	180	7.45 ± 1.36	7.25 ± 1.33	7.05 ± 1.39	6.75 ± 1.94	7.00 ± 1.41	7.30 ± 1.26	7.15 ± 1.27	7.05 ± 1.47
	Total	7.45^b^ ± 1.22	7.08^a^ ± 1.46	7.15^a^ ± 1.44	6.85^a^ ± 1.85	7.33^a^ ± 1.19	7.35^a^ ± 1.17	7.55^b^ ± 1.20	7.15^a^ ± 1.46
Total	160	7.28 ± 1.21	7.18 ± 1.24	7.40 ± 1.24	7.09 ± 1.36	7.34 ± 1.04	7.30 ± 1.10	7.40 ± 1.42	7.44 ± 1.10
	180	6.59 ± 1.43	6.71 ± 1.44	6.71 ± 1.38	6.48 ± 1.57	7.05 ± 1.21	6.96 ± 1.22	7.31 ± 1.31	6.93 ± 1.27
	Total	6.93 ± 1.36	6.94 ± 1.36	7.06 ± 1.35	6.78 ± 1.49	7.19 ± 1.14	7.13 ± 1.17	7.36 ± 1.36	7.18 ± 1.21

**Note:**

Values are presented in means ± standard deviation of 20 determinations. Mean values on the same column with different superscripts are significantly (*p* < 0.05) different. WFa = 100% wheat flour, WDFb = 90% wheat flour, 10% date flour, WDFc = 80% wheat flour, 20% date flour, WDFd = 70% wheat flour, 30% date flour.

#### Flavour and taste

In Table 6, the sensory score in flavour of cookies ranged from 6.83–7.08 for sample WDF_b_ (6.83) and WDF_d_ (7.08), respectively. There were no significant (*p* > 0.05) differences in flavour between samples baked at 160 °C and samples baked at 180 °C, although samples baked at 180 °C were having lower values. This could be attributed to the fact that at higher baking temperatures, the flavouring volatiles in the samples was more prone to lose. Also, the interaction between the two treatments (formulation and temperature) did not significantly (*p* > 0.05) affect the flavour as perceived by the panelist. Results obtained in flavour were higher than the values reported by Peter-Ikechukwu et al. (2017). An increase in flavour could be due to the effect of the pineapple juice that was used in the production of the cookies. Results obtained in the flavour of samples agreed with values (7.2–8.5) reported by Ade Kuldip, Lal & Mishra, (2015) in pineapple pomace powder fortified biscuits. Also presented in Table 6, the sensory scores in flavour of cookies ranged from 6.85–7.30 for sample WDF_b_ (6.85) and WDF_c_ (7.30), respectively. Significant (*p* > 0.05) differences were not observed in terms of taste for samples produced with different formulations, although an increase in baking temperature reduced scores for the taste. This agreed with the results reported by Peter-Ikechukwu et al. (2017) for wheat flour/date pulp-based cookies. In terms of the interaction between variation in the baking temperature and variation in the formulation, no significant (*p* > 0.05) interaction effect was observed. Further, the taste results of the cookies seem to agree with the data reported by Ade Kuldip, Lal & Mishra (2015).

#### Aftertaste and texture

The sensory scores in the aftertaste of the cookies ranged from 6.63–6.85 for sample WDF_b_ (6.63) and WDF_d_ (6.85), respectively. There were resembling (*p* > 0.05) among the samples. Temperature variation did not significantly affect the aftertaste despite the lower values at samples baked at 180 °C. High ratings for aftertaste may associate with processing temperatures unable to burn the samples. Moreover, the interaction between the processing temperature and samples obtained a significant effect (*p* < 0.05) specific to the aftertaste, which tends to reflect the opinion of Myers, Montgomery & Anderson-Cook (2009). In Table 6, the sensory scores in the texture of the cookies ranged from 6.95–7.33 for sample WF_a_ (6.95) and WDF_c_ (7.33), respectively. Thus, the increased substitution of wheat flour with date fruit flour occurred with texture, even though the mean scores across the samples resembled (*p* > 0.05). More so, the interaction between the processing temperature and the formulation also resembled (*p* > 0.05) by texture as rated by the panelists.

#### Mouth feel and crunchiness

In Table 6, the sensory scores for the mouthfeel of the cookies ranged from 6.93–7.35 with sample WF_a_ (6.93) and WDF_b_ (7.35), respectively. Significant (*p* > 0.05) differences were not observed between samples. The cookies baked at 160 °C were generally preferred by the panelist compared to the cookies baked at 180 °C. The mouthfeel is a sensation acquired when food is placed in the mouth and it is very important in sensory ratings for overall acceptability. High ratings by the panelist could be indicating a pleasant sensation of the cookies in the mouth. The interaction between the mixture formulation and the baking temperature was insignificant (*p* > 0.05). Table 6 also shows the sensory scores for the cookies crunchiness ranged from 6.85–7.63, significantly different (*p* < 0.05) between samples WF_a_ (6.85) and WDF_b_ (7.63). Both crispness/crunchiness are textural attributes often associated with freshness/firmness of natural manufactured foods (Tunick et al., 2013). The crunchiness was found to be highly noticeable, associated with pleasure and fun, regarded with warmth, and described as active, energetic, and appealing (Tunick et al., 2013). An inclusion of date fruit flour in the formulation increased with crunchiness of the cookies. In terms of crunchiness among the baking temperatures, the cookies baked at 160 °C were more preferred by the panelist compared to the cookies baked at 180 °C.

#### Overall acceptability

In Table 6, the overall acceptability of the cookies ranged from 7.00–7.30, with sample WF_a_ (7.00) as a minimum, and WDF_b_ (7.30) as a maximum. There was no significant (*p* > 0.05) difference between the treatment temperatures on the overall acceptability even though panelists gave higher values on cookies baked at 160 °C compared to samples baked at 180 °C. An increase in the baking temperature led to a decrease in the overall acceptability of the samples, which seemed to agree with the results observed by Patel et al. (2019) for de-oiled bottle gourd (*Lagenaria siceraria*) seed cake fortified biscuit. Formulation and processing temperature appeared not to affect overall acceptability (*p* > 0.05). Values for the overall acceptability were higher than the values reported by Peter-Ikechukwu et al. (2017) and within the range of values reported by Ade Kuldip, Lal & Mishra (2015) for pineapple pomace powder fortified biscuits. The sensory scores showed that sample WDF_b_ (7.30) was more preferred followed by sample WDF_c_ (7.28), then sample WDF_d_ (7.15), and WDF_a_ (7.00), respectively.

## Conclusions

Promising quality cookies can be produced from blends of wheat and date flour with pineapple juice. Date flour probably accounted for increased the ash, fibre, mineral, and carbohydrate content of the cookies. Cookies baked with 70% wheat and 30% date flour with pineapple juice had the highest nutrient retention. Cookies having a higher spread ratio could be more desirable, especially those baked with 90% wheat, and 10% date flour with pineapple juice. Also, cookies baked at 160 °C appeared more acceptable compared to the cookies baked at 180 °C. Studying the influence of different baking temperatures has a direct link with moisture/water, and therefore energy. Considering that the food, water, and energy nexus demands a broad scope, which should not be seen only to directly relate to climatic context, but should also be seen to indirectly relate to climatic context, the acceptability of cookies baked at 160 over those baked at 180 °C points to why the results of current work serve as a foundation further studies to specifically determine the energy requirements, and long-term environmental implications that this baking (temperature) position would pose.

## Supplemental Information

10.7717/peerj.14876/supp-1 Supplemental Information 1 Raw data.

## Additional Information and Declarations

### Data Availability

The following information was supplied regarding data availability: The raw data is available in the Supplemental File.
